# Regional analgesia for chest wall injuries related to cardiopulmonary resuscitation: retrospective service evaluation and injury pattern mapping*

**DOI:** 10.1002/anr3.70100

**Published:** 2026-09-09

**Authors:** J. M. Krawczyk, H. C. Hoskins, C. D. Rogers, K. Aassai Kannan, S. Aboalkaz, P. A. Carter

**Affiliations:** ^1^ Department of Anaesthesia University Hospital of Wales Cardiff UK; ^2^ School of Medicine Cardiff University Cardiff UK; ^3^ Radiology Department University Hospital of Wales Cardiff UK

**Keywords:** analgesia, cardiopulmonary resuscitation, chest wall, regional anaesthesia, rib fracture

## Abstract

Cardiopulmonary resuscitation frequently results in rib and sternal fractures. Regional analgesia is recommended in blunt chest trauma, but its role in injuries related to cardiopulmonary resuscitation is not well defined. This study aimed to characterise injury patterns and evaluate regional analgesia use in patients recovering from cardiac arrest. A retrospective service evaluation was undertaken at a tertiary centre over 2 years. Adults referred to the Acute Pain Service following return of spontaneous circulation were included. Data collected included patient characteristics, imaging, fracture distribution, STUdy of the Management of BLunt chest wall trauma (STUMBL) score, regional analgesia use and ventilatory requirements. Eighty‐one patients with chest wall injuries related to cardiopulmonary resuscitation (24% of all cardiac arrests) were referred. Rib fractures were identified in 84% and sternal fractures in 34% of patients; median STUMBL score was 38. Anterolateral fractures and anterior fractures were the most common. Referrals occurred a median of 2 days after the cardiac arrest. Regional analgesia was delivered to 40% of patients by the third day after the cardiac arrest. Fascial plane blocks predominated (92%), with technique selection influenced by operator expertise, fracture distribution and ease of insertion in the supine position. Analgesic effect was recorded as marked or moderate in 90%. Forty‐three per cent of the referrals were for patients who were mechanically ventilated, and 54% of them received regional analgesia. Chest wall injuries associated with cardiopulmonary resuscitation at our institution were common and frequently received regional analgesia. Injury patterns favoured anterior techniques, although prospective evaluation of effectiveness is required.

## Introduction

High‐quality cardiopulmonary resuscitation (CPR) requires chest compressions to a depth of 5–6 cm. The force required to achieve this frequently results in chest wall injuries. A recent meta‐analysis found that rib fractures occurred in approximately 55% (95% CI 48–62) of patients post‐CPR, with higher rates observed when automated compression devices were utilised [1].

Pain following blunt chest trauma is associated with impaired breathing, reduced cough efficacy and increased pulmonary complications. Management strategies are well described, with guidance including the vital role of regional analgesia (RA). Regional analgesia techniques reduce pain, improve respiratory mechanics and can reduce opioid requirements [2].

Patients recovering from cardiac arrest represent a distinct cohort within the blunt chest trauma population. Injuries predominantly involve the anterior and anterolateral aspects of the ribs and the sternum [1]. Patients after cardiac arrest are frequently sedated, mechanically ventilated, anticoagulated or neurologically impaired. All these aspects influence pain recognition, timing of intervention and choice of RA technique. The role of RA is not specifically addressed in current resuscitation or post‐cardiac arrest care guidelines [3]. As a result, decisions regarding recognition of pain, referral to specialist pain services and selection of RA techniques are variable and largely dependent on clinician expertise.

Available literature on RA in the post‐arrest group is limited, making it difficult to draw definitive conclusions regarding benefit. These uncertainties highlight the need for further structured evaluation. The aim of this study was to characterise injury patterns and evaluate the decision making, provision and timing of RA in patients recovering from cardiac arrest at a tertiary referral centre over a 2‐year period.

## Methods

We conducted a retrospective service evaluation at the University Hospital of Wales, Cardiff, which is a tertiary referral centre within the South Wales Major Trauma Network with 1060 inpatient beds and 36 adult critical care beds. Approval was granted by the institutional audit department. Data were anonymised and formal research ethics approval and individual patient consent were not required.

The study population was identified from a locally maintained Acute Pain Service (APS) database. Included patients were adults (≥ 18 years old) referred to APS following return of spontaneous circulation (ROSC) after an in‐hospital or out‐of‐hospital cardiac arrest between January 2024 and December 2025. The institutional resuscitation database provided denominator data for all cardiac arrests with ROSC during the same period. We excluded patients who received chest compressions without loss of cardiac output and those sustaining traumatic cardiac arrest.

Variables collected included patient characteristics; cardiac arrest location; computed tomography (CT) results; fracture distribution; STUdy of the Management of BLunt chest wall trauma (STUMBL) score [4]; timing of APS referral; timing, type and effectiveness of RA delivered; need for re‐site or technique modification; ventilatory requirements; and in‐hospital mortality. The perceived effectiveness of RA was evaluated by pain nurse specialists, who routinely perform structured follow‐up assessment for all patients referred to APS.

Following referral, all patients were reviewed by the acute pain team. Regional analgesia was considered in the context of elevated STUMBL score (> 21), persistent pain limiting respiratory function despite pharmacological optimisation, specific patient factors (obstructive sleep apnoea, frailty, chronic pain), significant injury identified on CT or failed tracheal extubation attributed to pain. Local anaesthetic bolus choice and dosing varied between clinicians and were not captured in this dataset. Continuous infusions followed a standardised institutional protocol of levobupivacaine 0.125% at up to 14 ml.h^−1^. Infusions were typically commenced at 10 ml.h^−1^ for the first 4 h where required to remain within a total maximum dose of 400 mg over 24 hours. The same total dose limits were applied when bilateral catheters were in situ. Regional analgesia catheters remained in situ for five to 7 days.

## Results

**Figure 1:**
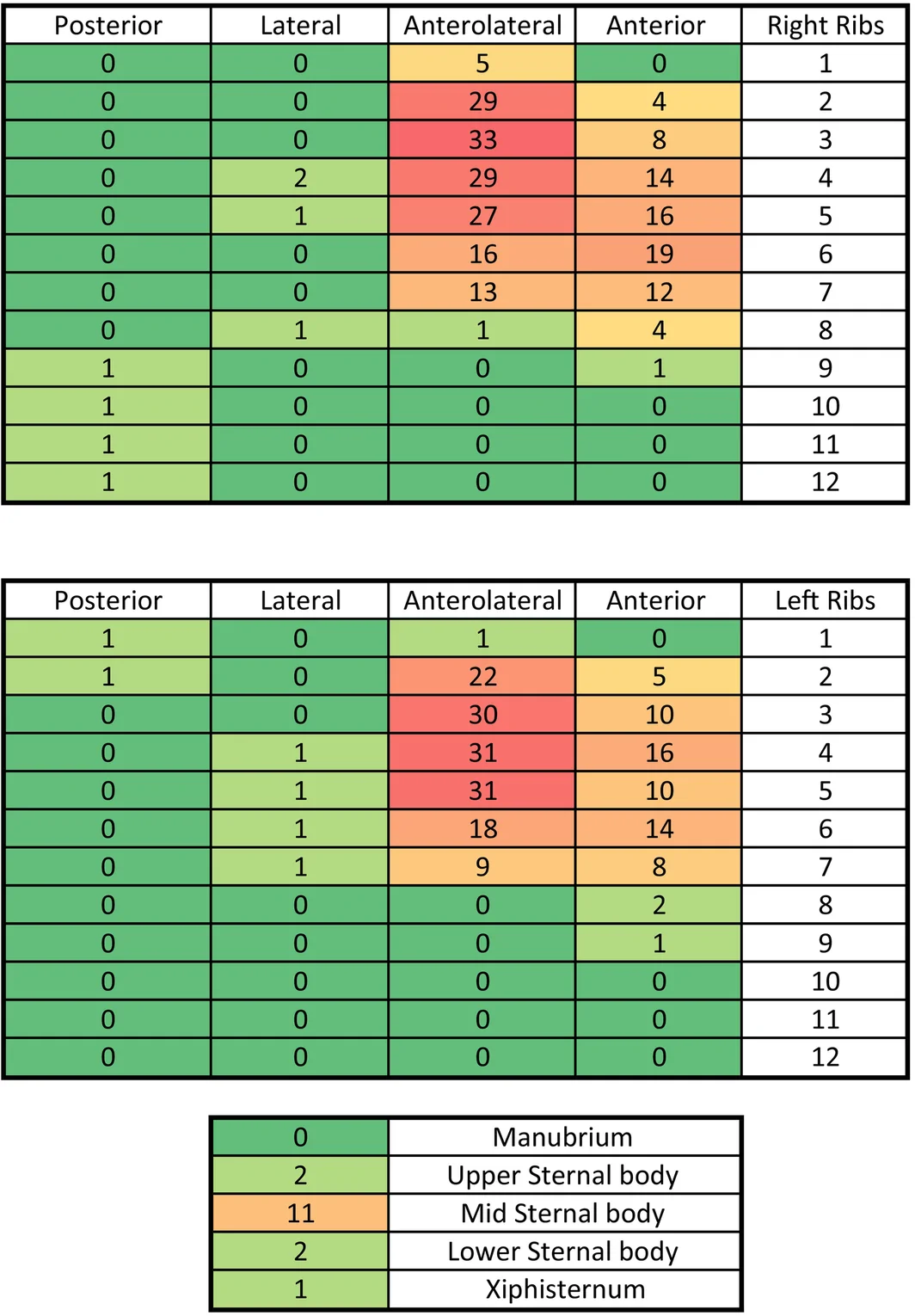
Distribution and frequency of acute rib and sternal fractures identified on computed tomography following cardiopulmonary resuscitation. A superimposed colour scale from red (highest frequency) to green (lowest frequency) depicts the density of fractures across rib segments. Fractures most commonly involved the anterolateral true ribs bilaterally and the mid‐sternal body, consistent with intended force distribution during chest compressions.

**Figure 2:**
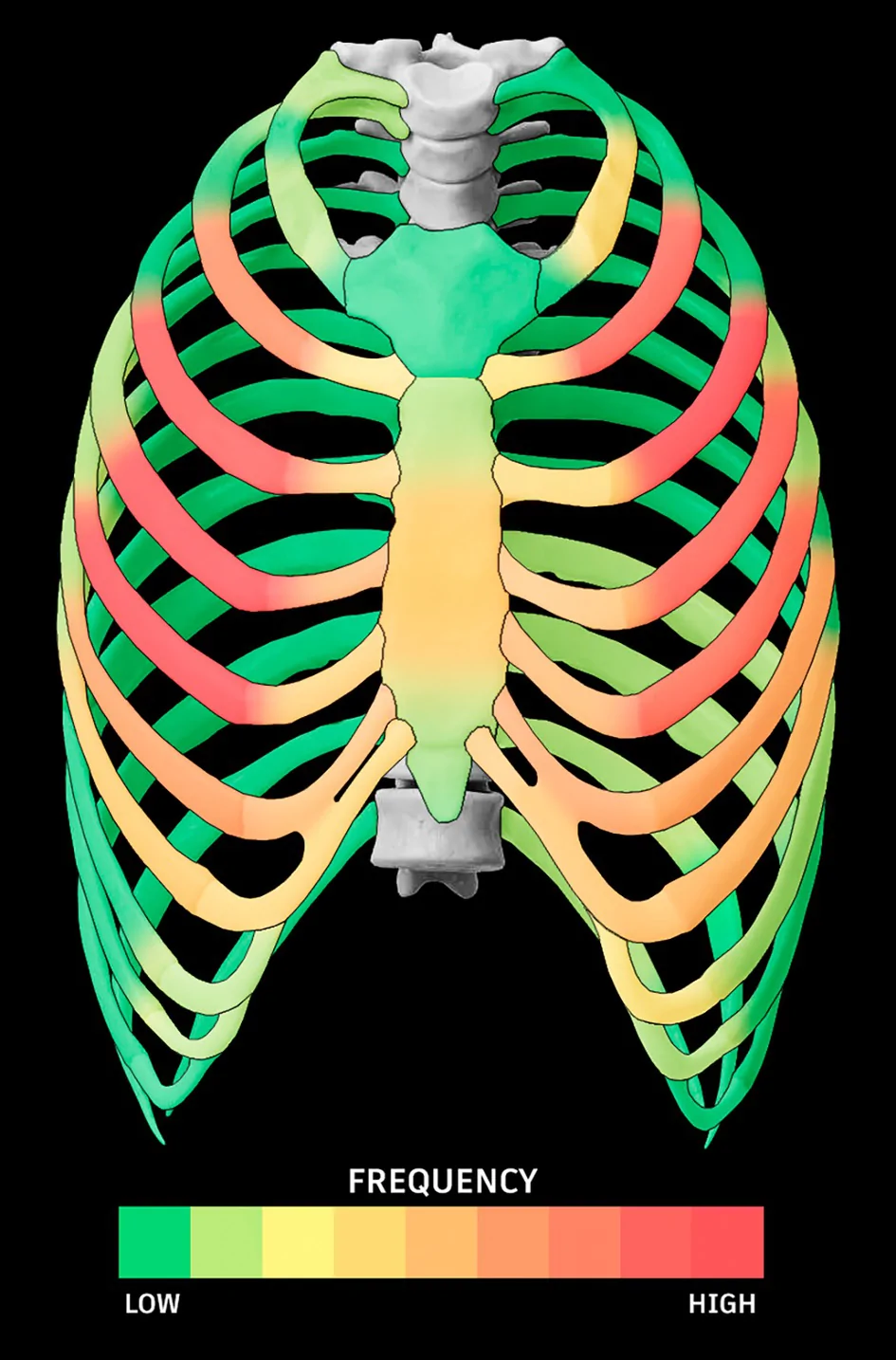
Heatmap of the rib cage demonstrating the collective frequency and anatomical location of acute rib and sternal fractures on computed tomography following cardiopulmonary resuscitation. Fracture density is displayed using a red‐to‐green colour scale, with red indicating the highest frequency and green the lowest.

Eighty‐one patients were referred to APS following CPR‐related chest wall injury, representing 24% of all cardiac arrests with ROSC during the study period. Median age was 67 years and 48/81 (59%) were male. Forty‐six patients (57%) sustained an out‐of‐hospital cardiac arrest and 35/81 (43%) had an in‐hospital cardiac arrest. In‐hospital mortality in the entire cohort was 19/81 (23%). Chest CT was performed in 58/81 (72%) patients. Rib fractures were present in 49/58 (84%) of imaged patients, while sternal fractures were reported in 20/58 (34%). Fracture distribution is shown in Figure 1 and Figure 2. The median STUMBL score was 38 (IQR 26–49).

Regional analgesia was delivered to 32/81 (40%) patients (Table 1). This represented 32/345 (9%) of all cardiac arrests during the study period. Median time from arrest to referral was 2 (IQR 1–3) days, and referral to RA was 1 (IQR 0–2) day. All procedures were performed either in the intensive care unit or in the theatre complex. The total number of regional blocks performed was 40. The majority of techniques were bilateral 27/40 (67%). Fascial plane blocks predominated 37/40 (92%), including erector spinae plane block (ESPB) 20/40 (50%), serratus anterior plane block (SAPB) 9/40 (22%) and superficial parasternal blocks 8/40 (20%). Three out of 40 (8%) patients received epidural analgesia.

**Table 1:** Regional analgesia provision, technique characteristics and repeat procedures in patients recovering from cardiopulmonary resuscitation.

Category	Value
**Regional analgesia provision**	
Patients receiving RA	32/81 (40%)
Institutional average RA rate for all rib fractures	57%
**Regional technique characteristics**	
Total RA procedures	40 (100%)
**Neuraxial techniques**	
Epidural	3/40 (8%)
**Fascial plane techniques**	37/40 (92%)
Bilateral blocks	27/40 (67%)
Unilateral blocks	10/40 (25%)
**Specific block types**	
Erector spinae plane block	20/40 (50%)
Serratus anterior plane block	9/40 (22%)
Superficial parasternal block	8/40 (20%)
**Repeat procedures**	
Second procedure required	8/40 (25%)
Catheter re‐site	3/8 (38% of repeats)
Change of technique	5/8 (63% of repeats)

RA, regional analgesia.

All RA techniques involved catheter placement to allow continuous local anaesthetic infusion. No complications related to RA were observed in the studied cohort.

Analgesic effect was reported as ‘marked’ in 25/40 (63%) of cases, ‘moderate’ in 11/40 (27%) and ‘minimal’ in 1/40 (3%). Effectiveness data were not available for 3/40 (7%) of patients. Eight patients of 32 (25%) required catheter re‐siting or change of technique. Of these, four were related to catheter dislodgement or technical issues and four involved a change of block type. In patients undergoing a change of technique, analgesia improved in two cases (erector spinae plane to parasternal, serratus anterior plane to epidural), remained suboptimal in one case (erector spinae plane to parasternal) and was not documented in one case (parasternal to serratus anterior plane).

Thirty‐five (43%) patients were receiving invasive mechanical ventilation at the time of referral (Table 2), of whom 19/35 (54%) received RA. Twelve of these (63%) were performed prior to tracheal extubation.

**Table 2:** Ventilation status and timing of regional analgesia in patients recovering from cardiopulmonary resuscitation.

Category	Value
Mechanical ventilation at time of referral	35 (43%)
Median duration of ventilation	3 days (IQR 2–7.5)
Ventilated patients receiving RA	19 (54%)
RA performed during ventilation	12 (63%)
RA after tracheal extubation	4 (21%)
Timing unknown	3 (16%)

RA, regional analgesia.

## Discussion

This service evaluation details the provision of RA for post‐CPR chest wall injuries at a tertiary hospital in the UK. In our cohort, 24% of post‐CPR patients were referred to the APS and 40% of those patients received RA. This contrasts with APS data from our institutional Major Trauma Centre annual report, where 57% of referrals for blunt chest trauma required RA. Differences may be explained by institutional factors, such as resource availability, ward capability for managing local anaesthetic catheters and pumps and clinician awareness that RA can be employed in this cohort of patients.

A median STUMBL score for the studied cohort was 38 (IQR 26–49). For context, RA in blunt chest trauma is routinely considered in our centre for patients with a score greater than 21. Ninety per cent of RA techniques performed were reported to have a ‘marked’ or ‘moderate’ improvement in patients' pain. The data presented here highlight a substantial demand and benefit of RA among post‐CPR patients, reinforcing the need for appropriate service provision in this area, as this patient group is likely to expand with improving cardiac arrest outcomes.

Iatrogenic chest wall injury following CPR is common, with reported rib fracture rates in approximately 55% and sternal fractures approaching 20% [1, 5]. Pain from rib fractures, particularly in patients with high STUMBL scores, is associated with impaired cough, hypoventilation and reduced mobility [6]. These factors increase the risk of hypoxia, retained secretions, pneumonia, venous thromboembolism, critical illness myopathy and chronic pain. Opioid‐based management may further compound respiratory compromise and delirium in the post‐arrest population.

The severity and trajectory of pain following CPR‐related rib fractures remain unclear. In contrast to patients with blunt chest trauma, post‐arrest patients are frequently sedated, mechanically ventilated or neurologically impaired following ROSC. Pain may therefore be under‐recognised or deprioritised amidst competing clinical concerns.

Referrals in our service evaluation were made directly by the treating clinical teams to the APS. Patients on the coronary care unit and general medical wards were typically referred when pain remained uncontrolled despite multimodal analgesia. A lower referral threshold was often observed in patients with pre‐existing chronic pain conditions. In the intensive care setting, referral patterns differed. Patients were referred when pain was considered to contribute to failure of tracheal extubation, ineffective cough or inadequate respiratory effort, or when chest wall injury was deemed clinically significant to warrant pre‐emptive RA before tracheal extubation.

The optimal timing of RA in the mechanically ventilated patients is uncertain. In our service evaluation, 63% of the patients requiring mechanical ventilation after cardiac arrest received RA before tracheal extubation. Blocks delivered before extubation may facilitate ventilator weaning, improve respiratory mechanics and potentially reduce sedation requirements [7]. Post‐extubation blocks allow clinical correlation of pain location to imaging, potentially permitting more precise RA techniques. A patient‐centred approach was employed at our institution depending on the documented injury and compounding factors present, such as respiratory morbidity and trajectory on weaning from mechanical ventilation. Additionally, it is unknown how long after injury regional techniques remain beneficial. The assessment of RA efficacy can also be influenced by underlying clinical factors, such as neurological recovery or respiratory reserve.

Fracture distribution following CPR differs from blunt trauma. While blunt injuries commonly involve the lateral and posterolateral aspects of ribs 4–7, CPR‐associated fractures predominantly affect the anterolateral segments [8]. This distinction has implications for regional technique selection and anterior blocks may be indicated. This is reflected in the RA techniques used at our institution, with serratus anterior and parasternal techniques making up 42% of RA techniques. In our cohort, CT identified fractures in 84% of cases, a higher fracture rate than typically reported in the literature [1, 5]. This discrepancy likely exists as our dataset represents patients referred to APS, a cohort likely to have more severe or uncontrolled pain who therefore had a greater probability of sustaining chest wall fractures.

Choice of RA technique in our centre varied according to the operators' role and experience. Erector spinae plane block and thoracic epidural analgesia were most commonly performed by on‐call anaesthetic teams. In contrast, serratus anterior plane and superficial parasternal blocks were predominantly performed by clinicians with specialist acute pain or RA expertise during dedicated acute pain sessions. Technique selection is influenced primarily by fracture distribution on imaging and, in patients requiring mechanical ventilation, the ability to perform the block in the supine position.

Posterior regional techniques include ESPB and thoracic epidural analgesia. The ESPB has become a routine block in rib fracture management due to its relative technical simplicity and favourable safety profile, particularly as an alternative to epidural or paravertebral block in patients on anticoagulation agents [9]. It is recommended as plan A block by Regional Anaesthesia‐UK [10]. A meta‐analysis of 37 studies reported significant reductions in pain scores with a low complication rate [9]. However, the extent to which ESPB reliably provides anterior chest wall analgesia remains debated. Cadaveric studies suggest variable anterior coverage [11, 12, 13], whereas imaging studies demonstrate inconsistent but sometimes extensive paravertebral and epidural spread [14]. Sensory block patterns may be unpredictable with ESPB, particularly for anterior rib and sternal fractures typical for patients who had had CPR. Bilateral catheter techniques are often required, but they may limit the available dose of local anaesthetic per side to avoid overdosing.

Thoracic epidural analgesia, once the gold standard for rib fracture analgesia [15], is often contraindicated in the post‐cardiac arrest population due to therapeutic anticoagulation or dual antiplatelet therapy for cardiac pathology. Although we did not capture anticoagulation and antiplatelet use in this dataset, this may partially explain the pattern observed in our findings: only 8% of RA techniques administered were epidurals, compared with 16% in traumatic rib fractures in our institution.

Anterior regional techniques, such as SAPB and parasternal blocks, are more likely to be indicated in CPR‐related injury due to the predominance of anterior and anterolateral fractures. Randomised trials in emergency department populations have demonstrated that SAPB can reduce pain scores and opioid requirements in patients with rib fractures [16]. Serratus anterior plane block and parasternal block can be performed in the supine position, making it particularly suited to patients requiring mechanical ventilation.

Pecto‐intercostal plane block was originally described in cardiothoracic and breast surgery and its application in sternal trauma remains poorly explored in the literature [17]. However, our experience demonstrates the application of the superficial parasternal approach in post‐CPR injuries. In the authors' opinion, this block is relatively simple to perform and because this technique provides coverage of the anterior cutaneous nerve branches, it may be especially useful when sternal fractures are accompanied by anterior rib fractures. Its effectiveness compared with other regional techniques remains unknown.

Practical considerations may limit the widespread implementation of RA in post‐cardiac arrest patients. Bilateral blocks frequently require dual infusion pumps or flow‐splitting devices, complicating dosing and monitoring. In addition, ward‐level expertise in managing local anaesthetic infusions varies. In our institution, patients requiring continuous regional analgesia cannot routinely be managed on general medical wards or the coronary care unit due to limited staff training; thus RA techniques often necessitate transfer to surgical or high dependency areas. Furthermore, RA techniques can be an obstacle to stepping down care from high dependency areas. Such system constraints may delay or preclude block provision and likely vary between centres.

This service evaluation represents the first examination of RA for CPR‐related chest wall injury, addressing a gap in literature largely focused on trauma. Using routinely collected data, it reflects real‐world practice in a heterogeneous post‐cardiac arrest population. However, it is limited by its single‐centre, retrospective design, small sample size and inclusion of only patients referred to APS, introducing selection bias. Outcomes were based mainly on clinician assessment rather than standardised pain scores. Data relating to chest infection were recorded inconsistently and so could not be included in the analysis. The observational design precludes any causal inference and observed practice was likely influenced by local expertise and infrastructure. As such, our service evaluation should be viewed as hypothesis‐generating and descriptive.

## Conclusions

Cardiopulmonary resuscitation‐related rib and sternal fractures constitute a distinct clinical scenario with unique anatomical, physiological and logistical considerations. Our findings demonstrate that RA can be successfully delivered in this cohort, including during mechanical ventilation, but practice is variable and influenced by institutional capability and system constraints.

While RA is beneficial in traumatic rib fractures, its role in CPR‐related injury remains uncertain. Prospective studies are required to determine optimal block selection, timing relative to tracheal extubation and impact on respiratory outcomes in patients recovering from cardiac arrest. Given the frequency of CPR‐related chest injury, targeted evaluation of analgesic strategies in this population is warranted.
